# TCR3d 2.0: expanding the T cell receptor structure database with new structures, tools and interactions

**DOI:** 10.1093/nar/gkae840

**Published:** 2024-09-26

**Authors:** Valerie Lin, Melyssa Cheung, Ragul Gowthaman, Maya Eisenberg, Brian M Baker, Brian G Pierce

**Affiliations:** Department of Cell Biology and Molecular Genetics, University of Maryland, College Park, MD 20742, USA; University of Maryland Institute for Bioscience and Biotechnology Research, Rockville, MD 20850, USA; University of Maryland Institute for Bioscience and Biotechnology Research, Rockville, MD 20850, USA; Department of Chemistry and Biochemistry, University of Maryland, College Park, MD 20742, USA; Department of Cell Biology and Molecular Genetics, University of Maryland, College Park, MD 20742, USA; University of Maryland Institute for Bioscience and Biotechnology Research, Rockville, MD 20850, USA; Department of Cell Biology and Molecular Genetics, University of Maryland, College Park, MD 20742, USA; University of Maryland Institute for Bioscience and Biotechnology Research, Rockville, MD 20850, USA; Department of Chemistry and Biochemistry, University of Notre Dame, Notre Dame, IN 46556, USA; Harper Cancer Research Institute, University of Notre Dame, Notre Dame, IN 46556, USA; Department of Cell Biology and Molecular Genetics, University of Maryland, College Park, MD 20742, USA; University of Maryland Institute for Bioscience and Biotechnology Research, Rockville, MD 20850, USA; University of Maryland Marlene and Stewart Greenebaum Comprehensive Cancer Center, Baltimore, MD 21201, USA

## Abstract

Recognition of antigens by T cell receptors (TCRs) is a key component of adaptive immunity. Understanding the structures of these TCR interactions provides major insights into immune protection and diseases, and enables design of therapeutics, vaccines and predictive modeling algorithms. Previously, we released TCR3d, a database and resource for structures of TCRs and their recognition. Due to the growth of available structures and categories of complexes, the content of TCR3d has expanded substantially in the past 5 years. This expansion includes new tables dedicated to TCR mimic antibody complex structures, TCR-CD3 complexes and annotated Class I and II peptide–MHC complexes. Additionally, tools are available for users to calculate docking geometries for input TCR and TCR mimic complex structures. The core tables of TCR–peptide–MHC complexes have grown by 50%, and include binding affinity data for experimentally determined structures. These major content and feature updates enhance TCR3d as a resource for immunology, therapeutics and structural biology research, and enable advanced approaches for predictive TCR modeling and design. TCR3d is available at: https://tcr3d.ibbr.umd.edu.

## Graphical Abstract

**Graphical Abstract:**
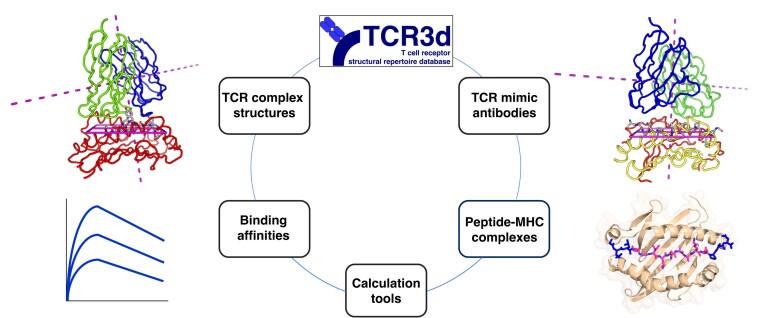

## Introduction

T cell receptors (TCRs) are heterodimeric immunoglobulin proteins found on the surface of T cells of humans and other vertebrates (1). They play a key role in cellular immunity by engaging foreign antigens, often in the form of peptides presented by major histocompatibility complex (MHC) proteins, leading to an immune response. Highly diverse repertoires of TCRs are present in each individual and can collectively bind to a vast array of viral and non-viral antigens. Due to the specific nature of their targeting and their role in T cell function, TCRs are increasingly being utilized as therapeutics for infectious disease and cancer (2,3), including in recent clinical studies (4,5). Aberrant TCR recognition can contribute to autoimmunity (6–8) and leads to the cellular rejection of transplanted tissues (9,10).

To enable insights into structures of TCRs and their recognition, in 2019 we released TCR3d (11), which is a regularly updated and annotated database containing all experimentally determined TCR structures from the Protein Data Bank (PDB) (12). TCR3d complements other databases devoted to TCR sequences (13,14) and affinities (15), and is distinguished from another database on TCR structures (16) by its content, annotations, and tools. In the past five years, the number and diversity of TCR and TCR-related structures have grown considerably, including those describing TCR recognition of cancer neoantigens (17) and SARS-CoV-2 epitopes (18–20), the architecture of the TCR-CD3 complex (21,22), as well as therapeutically relevant TCR mimic antibodies that engage peptide–MHCs (23,24). In conjunction with the expansion of experimentally determined structural data, deep learning tools are actively being developed to address major challenges, such as prediction of TCR–peptide–MHC complex structures or TCR specificity, often utilizing structural datasets from TCR3d or other databases for training or testing (25).

Here, we describe several major updates to TCR3d, made as a result of the growing classes of structurally characterized interactions, as well as to increase the general utility of the database. As noted below, these updates include: (i) new tables for classes of complexes (TCR mimics, TCR–CD3), (ii) annotations for peptide–MHC complexes and TCR–peptide–MHC affinities and (iii) interactive tools for binding parameter assessments.

## Materials and methods

### Structure identification and annotation

The pipeline to identify and annotate TCR chains and TCR–peptide–MHC complexes is the same as for the original TCR3d implementation (11). TCR chains are identified on a weekly basis from the PDB with hidden Markov model (HMM) based sequence searches. Hits are processed by scripts to identify and annotate TCR–peptide–MHC complexes, followed by manual inspection and processing if needed. TCR mimic antibody complexes with peptide–MHCs are likewise identified automatically from the PDB through identification of structures containing antibody and MHC chains, using HMMs representing antibody heavy and light chains. Peptide–MHC complexes are annotated with MHC allele through sequence comparison with IMGT reference MHC sequences (26), and peptide core residues for Class II peptide–MHCs are identified using NetMHCIIPan (27) and confirmed with structural analysis. TCR–peptide–MHC binding affinities for structures are from the ATLAS database (15) and originally obtained from the literature. In cases of multiple reported binding affinities measured for a complex (typically from different studies), a median value is used.

### Web site implementation

The TCR3d web site is implemented with Flask 3.0.3 and Python, with the Tabulator Javascript library used to generate tables, and plotly (https://plot.ly) used to generate interactive plots. The WebGL-based protein viewer PV (v.1.8.1) is used for structural visualization, and the database is implemented with SQLite.

## Results

### Expansion of existing content

**Figure 1.**
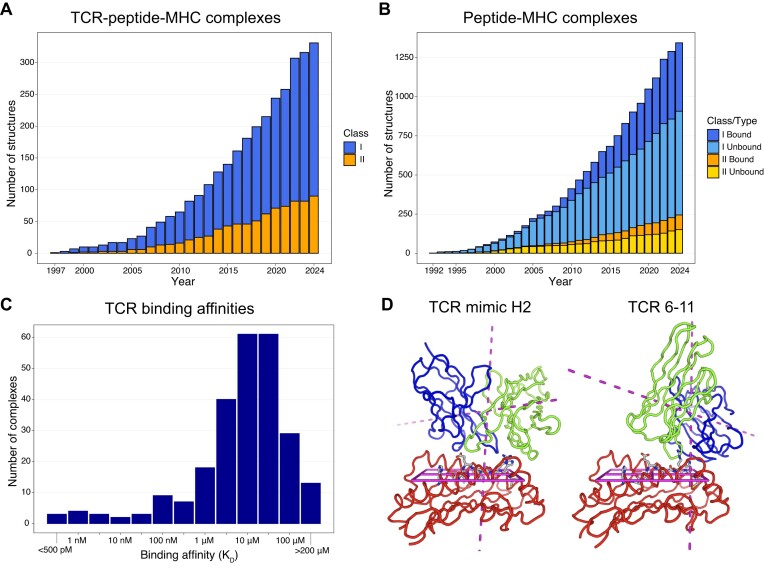
TCR3d content and updates. (**A**) Cumulative number of TCR–peptide–MHC complex structures in TCR3d by PDB release year, for Class I and II complexes. (**B**) Cumulative number of peptide–MHC complex structures in TCR3d by PDB release year, shown by MHC class (I or II) and whether the peptide–MHC is in a receptor-bound or unbound state. (**C**) Experimentally determined TCR–peptide–MHC binding affinities (*K*_D_s) annotated for structures in TCR3d, binned by affinity range and on a log scale. (**D**) A TCR mimic antibody, H2, in complex with peptide–MHC (PDB code 6W51; left) (23), and a human TCR, 6–11, in complex with the same peptide–MHC (PDB code 7RM4; right) (31), each shown the TCR3d structure viewer. Molecules are colored by chain, with antibody heavy chain and TCR α chain green, antibody light chain and TCR β chain blue, MHC red and peptide shown as gray sticks. The TCR inter-domain vector and inter-domain rotation axis are shown as dashed magenta lines, and the MHC helix plane is shown as a magenta grid.

Through regular updates from the PDB, the content of TCR3d has grown considerably since its original release, including its core Class I and Class II TCR–peptide–MHC complex tables. Currently there are 241 Class I complex structures and 90 Class II complex structures in TCR3d, compared with 153 and 62 structures for Class I and II, respectively, in 2019 (Figure 1A), representing an over 50% increase in available complexes.

### New tables and features

#### Class I and II peptide–MHC complexes

Tables representing all Class I and II peptide–MHC complexes in the PDB have been added to TCR3d. Each structure is annotated with the peptide sequence (including core subsequence for Class II epitopes), MHC allele, and whether the peptide–MHC is bound to a receptor or unbound. There are currently over 1330 peptide–MHC structures, with 1098 Class I and 235 Class II (Figure 1B).

#### Interactive tools

Web-based interfaces are available for calculating TCR–peptide–MHC engagement angles (crossing and incident angles) and TCR center of mass position over peptide–MHC for input PDB files. The center of mass calculations are offered for both angular coordinate (28) and Cartesian coordinate (29) formulations.

#### TCR binding affinities

To facilitate the development of TCR design and affinity prediction methods, experimentally measured TCR binding affinities have been added to the Class I and II complex structure tables for all complexes with reported affinities, and linked to the ATLAS database of TCR affinities and structures (15). This included identification of affinities from the literature for TCR–peptide–MHC complex structures that were released after the original ATLAS release in 2017. In total, 253 affinity values paired with complex structures are currently available. As expected, most are in the typical physiological range for TCRs (∼5–50 μM), however, there is a wide range in affinities overall, including over 30 complexes with affinities in the nanomolar range or higher (Figure 1C).

#### TCR mimic complexes and TCR–CD3 complexes

New tables are available containing structures of TCR mimic antibodies in complex with peptide–MHC targets, as well as TCRs in complex with CD3 proteins. TCR mimic antibodies are of increasing interest as therapeutics (30), and there are currently 16 TCR mimic complexes in TCR3d, most of which were deposited in the past 5 years. One of those complexes is shown in Figure 1D, with a TCR mimic antibody in complex with a cancer neoantigen from p53 and HLA-A2 MHC (23). For comparison, the TCR3d view for the separately determined structure of a human TCR targeting the same peptide and MHC (31) is also shown. Since the first cryogenic electron microscopy (cryo-EM) structure of a TCR-CD3 complex was released in 2019 (21), several more TCR–CD3 and TCR–CD3–peptide–MHC complex structures have become available; 13 of those complex structures are currently available in a dedicated table in TCR3d.

### Comparison with other databases

TCR3d is distinct from other available databases in its features and content. Several other databases feature TCR sequences with or without antigen specificity (13,14,32), but do not contain structural information. There are two other available databases that, like TCR3d, are focused on available structures of TCRs or their targets. STCRDab (16) contains structures of TCR chains and complexes, as with TCR3d, but distinctions of TCR3d include geometric parameters on TCR–peptide–MHC engagement (crossing angle, incident angle) for all structures, a large set of measured TCR–peptide–MHC affinities available for structures, and sets of all known peptide–MHC structures. Another structure-focused database, HLA3DB, has recently become available (33). While it includes useful annotations of structures of peptide–MHC complexes from the PDB, it only contains structures with human MHC alleles, and does not include Class II peptide–MHC complexes, whereas TCR3d contains mouse and other non-human MHC alleles as well as Class II peptide–MHC complexes. Furthermore, HLA3DB does not contain structures of TCRs or their recognition. The Immune Epitope Database (IEDB) contains structural information linked to entries retrieved through its epitope search interface (34), but without dedicated tables of TCR complexes and their annotations as in TCR3d.

## Discussion

TCR3d continues to provide a regularly updated and accessible view of structures of TCRs and their recognition to the community. This resource can be used as a reference for comparison of new experimentally determined complex structures through its datasets or calculation tools, as in previous studies (18,23,31,35). With recent exciting developments in deep learning to model and design protein interactions (36,37), there is a major need for comprehensive datasets and benchmarks to train and assess methods for systems of interest. In this regard, the structural data in TCR3d can enable development of approaches to model TCR–peptide–MHC complex or peptide–MHC structures from sequence, or to perform TCR specificity prediction, which despite recent advances (38–42) remain major important challenges for the community (25,43). Those interested in structure-based design of TCRs or immune receptors in general can utilize the recently added complex affinity data, while the peptide–MHC complex structures may be of interest as targets for prospective TCR design studies. By continuing to expand TCR3d, we aim to support the community in addressing these and other challenges in immunology research.

## Data Availability

TCR3d is freely available at: https://tcr3d.ibbr.umd.edu. The code for TCR docking angle calculations is available on Github (https://github.com/piercelab/tcr_docking_angle) and Zenodo (https://doi.org/10.5281/zenodo.13750762).
